# Comparing Dutch Case management care models for people with dementia and their caregivers: The design of the COMPAS study

**DOI:** 10.1186/1472-6963-12-132

**Published:** 2012-05-28

**Authors:** Janet MacNeil Vroomen, Lisa D Van Mierlo, Peter M van de Ven, Judith E Bosmans, Pim van den Dungen, Franka J M Meiland, Rose-Marie Dröes, Eric P Moll van Charante, Henriëtte E van der Horst, Sophia E de Rooij, Hein P J van Hout

**Affiliations:** 1 Department of Internal Medicine, Section of Geriatric Medicine, Academic Medical Center, University of Amsterdam, Amsterdam, Netherlands; 2 Department of Psychiatry, EMGO Institute for Health and Care Research, Alzheimer Center, VU University Medical Centre, Amsterdam, Netherlands; 3 Department of Nursing Home Medicine, EMGO Institute for Health and Care Research, Alzheimer Center, VU University Medical Centre, Amsterdam, Netherlands; 4 Department of Epidemiology, and Biostatistics, VU University Medical Centre, Amsterdam, Netherlands; 5 Department of Health Sciences and EMGO Institute for Health and Care Research, Faculty of Earth and Life Sciences, VU University Amsterdam, Amsterdam, Netherlands; 6 Department of General Practice, Academic Medical Center, University of Amsterdam, Amsterdam, Netherlands; 7 Department of General Practice and Elderly Care Medicine, VU University Medical Centre, EMGOInstitute, Amsterdam, Netherlands

**Keywords:** Dementia, Case management, Design, Clinical evaluation, Economic evaluation, Process evaluation, Informal caregivers, Implementation

## Abstract

**Background:**

Dementia care in the Netherlands is shifting from fragmented, ad hoc care to more coordinated and personalised care. Case management contributes to this shift. The linkage model and a combination of intensive case management and joint agency care models were selected based on their emerging prominence in the Netherlands. It is unclear if these different forms of case management are more effective than usual care in improving or preserving the functioning and well-being at the patient and caregiver level and at the societal cost. The objective of this article is to describe the design of a study comparing these two case management care models against usual care. Clinical and cost outcomes are investigated while care processes and the facilitators and barriers for implementation of these models are considered.

**Design:**

Mixed methods include a prospective, observational, controlled, cohort study among persons with dementia and their primary informal caregiver in regions of the Netherlands with and without case management including a qualitative process evaluation. Inclusion criteria for the cohort study are: community-dwelling individuals with a dementia diagnosis who are not terminally-ill or anticipate admission to a nursing home within 6 months and with an informal caregiver who speaks fluent Dutch. Person with dementia-informal caregiver dyads are followed for two years. The primary outcome measure is the Neuropsychiatric Inventory for the people with dementia and the General Health Questionnaire for their caregivers. Secondary outcomes include: quality of life and needs assessment in both persons with dementia and caregivers, activity of daily living, competence of care, and number of crises. Costs are measured from a societal perspective using cost diaries. Process indicators measure the quality of care from the participant’s perspective. The qualitative study uses purposive sampling methods to ensure a wide variation of respondents. Semi-structured interviews with stakeholders based on the theoretical model of adaptive implementation are planned.

**Discussion:**

This study provides relevant insights into care processes, description of two case management models along with clinical and economic data from persons with dementia and caregivers to clarify important differences in two case management care models compared to usual care.

## Background

Dementia is a chronically and devastating disease marked by memory loss and other cognitive impairments as well as behavioural lapses resulting in pronounced consequences for the people with dementia, their families and society. The cost of care for dementia patients, living at home or in a nursing home/home for the elderly, comprised 4.7% (€3.2 billion) of the total health care expenses in 2005 making it one of the most costly diseases in the Netherlands [1,2]. The number of people with dementia is expected to steadily rise due to an ageing population. In the World Alzheimer Report 2009, Alzheimer Disease International estimated that 36 million people worldwide live with dementia, with numbers doubling every 20 years to 66 million by 2030, and 115 million by 2050 [2,3]. The total mean duration of the disease from diagnosis until death was estimated in a large Dutch, prospective, observational analysis of a dementia nursing home cohort to be seven years [4]. Study participants lived in the community for on average five years and then in a nursing home for two years. The median life expectancy from the start of the dementia symptoms varied from 10.7 years for the 65–69 year olds to 3.8 years for those over 90 years old [3]. In a British, population-based cohort of 1304 elderly participants the estimated median survival time from dementia onset to death was 4.1 years for men and 4.6 year for women [5].

In the Netherlands, approximately 70% of the persons with dementia live in the community; a situation that is financially stimulated by current health policies [4,6]. During the course of the disease, persons with dementia become more and more dependent on others, mainly informal caregivers, such as spouses or children. Caring for a person with dementia is a burdensome task for informal caregivers and is associated with more frequent visits to health care professionals and more mental health problems [7-12]. Many services are available to effectively support people with dementia and their caregivers such as several types of daycare and respite care, psychoeducation, discussion groups, telephone support systems Alzheimer cafés and the Meeting centers support programme [13,14]. Specialized home care help to support people with dementia in (instrumental) activities of daily living, memory training and cognitive stimulation. Because of the multiplicity and complexity of problems that persons with dementia and their carers encounter in daily life, comprehensive and combined support programs for persons with dementia and their caregivers were developed in addition to the single interventions. In general, these combined multifaceted support programs are found to be more effective than single interventions in improving the mental health of persons with dementia and/or the carers and in delaying admission to long stay care facilities [15-17]. Furthermore, it is shown that unfulfilled needs of people with dementia are correlated to increased rates of nursing home admission and death [18].

With this wide variety of services available for people with dementia and carers, people often lack information regarding the available services that address their needs and many experience insufficient alignment, management and continuity of care and support during the disease trajectory [19-21]. It is essential to have a strong collaboration between various disciplines in the care and support sector involved in the management of dementia patients. In the past decade, several case management programmes were developed in the Netherlands to address this need. Case management is defined as “a collaborative process in which a case manager assesses, plans, implements, co-ordinates, monitors and evaluates the options and services required to meet an individual’s health, social care, educational and employment needs, using communication and available resources to promote quality cost effective outcomes” [22]. It strives to provide pro-active care coordinated by a case-manager who is supported by a multidisciplinary team of providers of elderly care (e.g. psychologist, geriatrician, general practitioner, neurologist or psychiatrist). The chronic care model (CCM) [23,24] and the guided care model [25] contain quality components that provide a basis for more coordinated care. This is helpful for heterogeneous chronic care trajectories like those found in dementia. Many case management organisations in the Netherlands are modelled after the chronic care model. Models based on CCM consist of a treatment hierarchy starting with specific and anticipatable care for patients with well-defined, uncomplicated problems up to case management which is reserved for patients with highly complex problems who have unpredictable care needs [25]. In this context, it is questionable whether case management is needed for all dementia severity levels. Based on the CCM, healthcare providers should distinguish between more and less complex patients and design cost effective stepped care models including case management for complex cases as recommended by several studies [23,24,26]. This was also demonstrated by Jansen et al. 2011 [27], who found that early detected persons with modest dementia behavioural problems demonstrated no benefit from case management. We aim to generate evidence on the cost effectiveness of case management in comparison with usual care while taking into account levels of behavioural complexity.

Reviews and meta-analyses of case management have yielded inconsistent results on clinical outcomes such as caregiver burden and depression and on economic outcomes [28-31]. Furthermore, only short term effects on caregiver satisfaction were found [32]. Only one meta-analysis including 15 studies found that risk of institutionalization of dementia patients was reduced by multicomponent interventions aimed at caregivers [28]. Based on this meta-analysis, clinicians are advised to tailor interventions according to the specific needs of the individual caregivers and care receivers [28]. Several reviews have indicated that further research should distinguish subpopulations that could benefit from case management [28-30].

In the Netherlands, patients and caregivers are highly satisfied with case management [33]. However, various models of case management are implemented, differing with respect to how services are delivered and by whom, the training provided to its staff and the means of financing. It is unknown whether there are also differences in clinical and cost-effectiveness between these case management models compared with usual care. In the COMPAS study, two different case management models are studied, that is the linkage model and the intensive case management/joint agency model. These models will be compared with usual care and with each other (description in Table 1). These case managements models were chosen based on their emerging prominence in Dutch healthcare for a number of years. The objective of this study is to identify differences in clinical, process and cost outcomes for people with dementia and informal caregivers between the two case management models and the usual care model. Furthermore, the aim is to identify facilitators and barriers in the implementation of the two case management models. This process evaluation focusses on which aspects are successful and their pitfalls.

**Table 1:** Comparison of case management models and care as usual

**Characteristics of different models**	**Linkage Model**	**Intensive Case management/joint agency model**	**Care as usual**
Central point for registration of cognitively impaired	New clients are referred by GP or health specialist to the central registration point	New clients are referred by GP or health specialist to the Multidisciplinary team at central registration point	No
Delivery of services	Independent services and networks	Mainly offered by one organization	Various networks
Possibility to diagnose dementia	By GP or referral to e.g. memory clinic or elderly care physician of mental health care service	By GP or by Multidisciplinary team after referral to central registration point	By GP or referral to memory clinic or elderly care physician of mental health care service
Case manager/dementia nurse	Yes	Yes	nurse present, incidentally
Social psychiatric nurse	No	No	Possible
Multidisciplinary team	External team that case managers can consult as per required	Case manager, elderly care physicians, neuropsychologist, neurologist, geriatrician, psychiatrist, dementia consultant all work within the same organisation as the case managers	No

Our first hypothesis is that in the two case management models the persons with dementia will have less behaviour and mood problems than persons receiving usual care and informal caregivers experience better mental health compared to usual care. Furthermore, we expect a delay in nursing home admission for persons with dementia versus usual care because care needs are better monitored and followed up by services that cooperate within a chain of care. Additionally, an exploratory hypothesis is whether the intensive care/joint agency model is associated with fewer behavioural problems and better follow up care compared to the linkage model, as the services that are part of one organisation may generate more efficiency.

## Methods

### Mixed method study design

A prospective, observational, controlled, cohort design to evaluate clinical and economic effects is accompanied by a qualitative process evaluation for the study on facilitators and barriers of implementation of case management. For the controlled cohort study, dementia care-receivers and their primary informal caregiver in different case management models are compared with those from regions that do not offer case management. The participants are recruited into the study and assessed every six months for two years. The Medical Ethics Committee of the VU University medical center approved the study protocol.

### Care models

The case management models that are key in this study in this project, as well as the care as usual, are described in detail below.

### Linkage model

The linkage model is comprised of independent care agencies where case managers provide the caregiver and patient with informative, practical, and emotional support [34]. In the linkage model, the introduction of case management care starts directly after the patient receives the diagnosis of dementia [35]. The case manager assesses the need for acute help during a telephone intake interview [35]. During an interview at the patient’s home a care and support plan is established that encompasses problems, needs, goals and interventions [22]. Case managers facilitate care by referring patients and their caregivers to various providers of health, social or community care until time of nursing home admission or death of the patient [35]. The case manager implements and monitors the care and support plan under the supervision of an elderly care physician and is in constant communication with the patient’s social system. In addition, during regular meetings with an elderly care physician, guidance is provided for case managers [35].

### Intensive case management model and joint agency model

The second case management model under study is a mix of two existing models: the intensive case management model and joint agency model [34]. Case managers in the intensive case management model guide and support people with dementia for long periods of time and provide care services within one organization [34]. In the joint agency model, the case manager is supported by a multidisciplinary team that includes different health specialists (Table 1) [34]. In this mixed model, the case management process starts with a referral from the general practitioner or a specialist requesting a diagnosis for possible cognitive impairment or (type of) dementia [35]. After the assessment, which is predominantly based on the DSM-IV diagnostic criteria, a care plan is created by the case manager together with the informal care giver(s) and patient [35]. The care plan includes problem and needs areas, intervention possibilities and yearly goals [35]. The care plan is communicated with the general practitioner. Implementation of the care plan is done by the case manager who also monitors it regularly. Like the linkage model, the patient and their family are supported by the case manager until nursing home admission or death of the patient [35].

### Usual care

Care as usual is defined in this study, as “care that is primarily provided or coordinated by the general practitioner and, as a rule, does not include access to a case manager” [36]. Usual care is care according to the guidelines for dementia patients from the Dutch College of General Practitioners [36]. Care may be monitored by a nurse working in general practice in addition to the general practitioner. Patients have access to community services such as home care, day care or meeting centers for people with dementia and their caregivers. A social psychiatric nurse from mental health services is sometimes available for people with dementia that have severe behavioural problems.

### Participants and setting for the controlled cohort study

Pairs of persons with dementia and their primary informal caregiver are included in the study. Persons with dementia are eligible for this study if they live at home, have a diagnosis of dementia, are not terminally-ill, are not anticipated to be admitted to long term care facilities within 6 months, and have an informal caregiver. The informal caregivers are eligible if they are the primary informal caregiver responsible for looking after the patient, have sufficient language proficiency and are not severely ill. Persons with dementia and their caregivers will be recruited from various regions in the North, West and Center of the Netherlands that comprise both rural and metropolitan areas.

### Procedure

The COMPAS study plans to recruit in the experimental group individuals diagnosed with dementia and their caregivers receiving case management from different regional case management models in the Netherlands. Case managers provide lists of patients who are eligible to participate. Potential participants receive written information about the project either via a letter from the research coordinator or via the case manager who approaches them with information directly, asking them if they agree to be contacted by a researcher with more detailed information on the study. Potential participants can either mail (a pre-stamped envelope is attached to the written information) or call the research coordinator or the case manager to let him/her know that they are interested in this additional information. Subsequently, the interviewers call the potential participants to inform them about the study in more detail and to set an appointment for the baseline interview with those who are willing to participate. Interviewers also call participants who have not replied within two weeks after the letter is sent to see if they are interested to participate in the study. For the usual care participants, who do not receive case management, recruitment occurs via outpatient geriatric or neurologic (memory) clinics, Alzheimer centers and general practices where a letter is sent to ask them to participate followed by a phone call from the interviewers to ensure that the letter was received and to provide more information on the study.

Prior to the interviews, all participants will sign an informed consent form. Patients and caregivers are interviewed at their homes by trained research interviewers using case record forms. The cost diary is left at the house, collected during the next interview session, and a new cost diary is provided for the upcoming six months. All outcomes are measured at baseline, 6, 12, 18 and 24 months via questionnaires and interviews. Information on the dementia diagnosis is gathered from the informal caregiver. The caregiver is asked when first symptoms were detected and time of diagnosis. Recruitment started in March 2011. All people currently in case management are screened for eligibility and asked to participate. Data collection is expected to end in 2014.

### Measuring instruments

#### Primary clinical outcome measures

The primary outcome variables are 1) the frequency and severity of behavioural problems of the person with dementia as assessed by the Neuropsychiatric Inventory (NPI) [37] and 2) psychological stress of the primary informal caregiver as measured by the General Health Questionnaire (GHQ-12) [38]. The NPI includes 12 domains which measure severity (3-point scale) and frequency (4-point scale) of behavioural and psychological symptoms of the patients as well as the emotional impact of the symptoms on the caregivers. Analyses of the psychometric properties of the original ten-item version showed high internal consistency reliability for the frequency*severity product scores (Cronbach’s α = 0.88) and for the specific severity (α = 0.87) and frequency (α = 0.88) ratings [37,39]. The NPI was chosen as the primary outcome in this study because behavioural problems often precede the more relevant cost-carriers like crisis management or institutionalization [40-43] and case management is expected to positively impact behavioural problems and the stress informal caregivers experience. The GHQ-12 was chosen as a primary outcome measure because it is a general measure of mental health and mental health complaints of the caregiver being an important predictor of institutionalization for the person with dementia [40]. The GHQ-12 comprises of three domains: social dysfunction, anxiety and loss of confidence [44]. The scoring method is a four-point response method with excellent reliability (Cronbach’s alpha = 0.90) [44]. Table 2 presents an overview of the primary and secondary clinical outcomes measures for the persons with dementia and their caregivers.

**Table 2:** Outcome measures in the COMPAS study

**Patient**		**Concept**	**baseline**	**6 months**	**12 months**	**18 months**	**24 months**
Primary outcomes	Neuropsychiatric inventory [37]	Behavioural and psychological symptoms *	X	X	X	X	X
Secondary outcomes	Quality of Life in Alzheimer’s Disease [45]	Quality of Life**	X	X	X	X	X
	Short Form-12 [46]	Health related Quality of Life*	X	X	X	X	X
	EuroQol- 5D + C [47]	Health Related Quality of Life**	X	X	X	X	X
	Camberwell assessment of needs for the elderly (CANE) [21,48]	Needs**	X	X	X	X	X
Cost outcomes	Cost diary	Economic evaluation	X	X	X	X	X
	Time between diagnosis and institutionalization	Economic evaluation	X	X	X	X	X
	Time between first symptoms and institutionalization	Economic evaluation	X	X	X	X	X
Effect modifiers or confounders	Age		X				
	Gender		X				
	Marital status		X				
	Living accommodation	Living alone or with someone	X				
	Education		X				
	Mini Mental State Exam [49]	Cognition	X		X		X
	KATZ Activities of Daily Living-5 [50]	Activities of daily living*	X	X	X	X	X
	Co-morbidity		X				
	Dementia type		X				
	Time in case management		X				
Caregiver							
Primary outcomes	General Health Questionnaire −12 [38]	Emotional stress mental health complaints	X	X	X	X	X
Secondary outcomes	Short Sense of Competence Questionnaire SSCQ [51]	Perceived care competence	X	X	X	X	X
	Pearlin Mastery Scale [52]	Self-efficacy	X	X	X	X	X
	EuroQol 5D[53]	Health related quality of life	X	X	X	X	X
	Carer-Qol [54]	Health related quality of life	X	X	X	X	X
	SF12 [46]	Health related quality of life	X	X	X	X	X
Cost outcomes	Informal care	Economics evaluation	X	X	X	X	X
Quality of care	Quality indicators [43,55]	Process of Care	X	X	X	X	X
Effect modifiers or confounders	Age		X				
	Education		X				
	Relation to person with dementia		X				

*by proxy, **patient and proxy.

#### Secondary clinical outcomes

Secondary outcome variables for the patient include the following. First, Mortality and institutionalization data will be collected from the informal caregiver or general practitioner. Generic Quality of life will be measured by the SF-12 [46] and the EQ-6D (EuroQol) [55]. Next to that, disease specific quality of life will be measured using the Qol-AD (Quality of life in Alzheimer’s Disease) [45] and EuroQoL-6D all quality of life questionnaires will be completed/administered to the care receiver as well as the primary caregiver proxy. Number of met and unmet needs based on the Camberwell assessment of needs for the elderly (CANE) [21,48] administered to the care receiver as well as the primary caregiver proxy. Number of crises defined by emergency department visits, unplanned hospitalization and unplanned institutionalization through the cost diary.

Secondary outcomes variables for the informal caregiver include:

(1) Sense of competence to care measured by the Short Sense of Competence Questionnaire (SSCQ)(Cronbach’s α = 0.76) [51]

(2) Empowerment measured by the Pearlin Mastery scale [52].

(3) Quality of life measured by the Short Form 12, CarerQol [54] and EuroQoL-5D [53].

(4) Number of experienced crises defined by emergency department visits, unplanned hospitalization and unplanned institutionalization.

### Quality of care outcome measures

Quality of care at the micro level of the care receivers is measured by:

(1) 12 indicators on treatment, education, support, and safety (translated and back-translated) from Vickrey asked to the informal caregiver, such as care plan developed, behavioural problems discussed, and non-pharmacological treatments [43];

(2) 10 Indicators from the problems and needs questionnaire, developed by the Dutch Alzheimer Association in conjunction with the Netherlands Institute for Primary Care Research (NIVEL), asked to the informal caregivers [27].

### Cost outcome measures

Cost diaries are used to collect data on use of care and support and direct and indirect healthcare and non-healthcare costs for patients and caregivers to estimate costs from a societal perspective. Direct healthcare costs include formal care such as general practitioner visits and medication use. Direct nonhealthcare costs consist of time spent to care by informal carers. Indirect health care costs include medical costs in life years gained. Indirect non-healthcare costs include days absent from paid work or unable to do daily activities such as housekeeping or voluntary work for caregivers.

### Process outcome measures

For the qualitative process evaluation, the semi-structured interviews with key figures regarding facilitators and barriers of implementation of case management will be based on the theoretical model of adaptive implementation [56,57]. This model distinguishes different phases of implementation (preparation-, implementation- and continuation phase), and describes factors that can influence implementation at the micro- (care-provider, patient and informal carer), meso- (collaboration between care providers/organizations) and macro-level (legal and financial framework) for each phase. The interviews are conducted with case managers, project leaders, care coordinators as well as insurers, client organisations and municipalities until a saturation point is reached. An extensive description of the different case management approaches will be made through data collected by a questionnaire that is based on the Chronic Care model and the essential components of case management as described by Verkade et al. [58].

### Sample size

The sample sizes for the controlled cohort study are calculated based on the primary outcome measures NPI [37] and the GHQ-12 [38]. Based on Callahan et al. [42], who investigated the effect of collaborative care, the estimated change between baseline and follow-up after 12 months on the NPI was −2.5 (SD 13.7) in the intervention group and 2.7 (SD 20.3) in the control group, resulting in a difference of −5.2. The standard deviations reported are the averages for the two time points. Standard deviations of baseline and follow-up scores were assumed equal when calculating the required sample size. The NPI has moderate to high test - retest reliability scores therefore we assumed a within-subject correlation of 0.5 to estimate the standard deviation for the difference scores based on the standard deviations of the baseline and follow-up [11]. A sample size of 140 participants per group is considered sufficient (80% power) to detect a difference of 5.2 in change scores between the groups. This calculation was based on a two group Satterthwaite *t*-test at a one sided significance of α = 0.05. To allow for a drop-out rate of 20% (including patients who withdraw or die in the course of the study), 175 (140/0.80 = 175) participants per group will be recruited.

Based on a study by Dias et al., the difference in GHQ baseline and follow-up at six months was −1.4 (SD 2.8) for the caregiver intervention group and 0.8 (SD 3.4) in the control group [59]. The GHQ has moderate to high test-retest reliability scores. A within-subject correlation of 0.6 when estimating the standard deviation of the difference scores was used. Using a two group Satterthwaite *t*-test with a onesided significance level of α = 0.05, an initial sample size of 21 in each group was estimated to have 80% power to detect a difference in means of −2.2 on the GHQ. A dropout rate for caregivers was assumed at 20% which resulted in a final sample size of at least 26 caregivers per group [59].

### Data analyses

Demographic, clinical and prognostic characteristics of the study participants will be collected and described in detail. The number of participants with missing data for each variable of interest will be provided along with an analysis to check for selective missingness. Average follow up time will be determined for each model. Reports on adjusted and unadjusted values with confounders will be presented along with 95 percent confidence intervals. Confounders that change beta coefficients more than 10% will be reported.

### Effect evaluation

Data will be analyzed by the intention to treat principle. Propensity score techniques will be used to address potential selection bias [60]. The propensity score is a one-dimensional summary measure that is directly related to the predicted probability that a subject with specific baseline characteristics is included in the intervention group (relative to the control group) [60]. Propensity scores are derived as the fitted linear predictor values from a logistic regression model with treatment (per case management model versus usual care) as the outcome variable. All variables on which groups differ at baseline are included as covariates. Depending on the mismatch of the intervention and control group on these propensity scores we will use 1-to-1 matching, stratified (quintile) matching or choose to include the propensity score as a confounder in the regression models for treatment effects [61].

### Economic evaluation

A societal perspective is used in this economic evaluation. Missing data will be imputed using multiple imputation. As patient/caregiver-level cost data have a highly skewed distribution, bootstrapping will be performed to estimate Approximate Bootstrap Confidence (ABC) intervals around cost differences [62,63]. Incremental cost-effectiveness ratios (ICERs) will be calculated by dividing the difference in total costs between the case management models and usual care by the difference in clinical effects. Non-parametric bootstrapping will also be used to estimate the uncertainty surrounding the ICERs (5000 replications). The bootstrapped cost-effect pairs will be plotted on a cost-effectiveness plane (CE plane) [64] and used to estimate cost-effectiveness acceptability curves (CEA curves). CEA curves illustrate the probability that the intervention is cost-effective in comparison with the control treatment for a range of ceiling ratios. The ceiling ratio is defined as the societal willingness to pay in order to gain one unit of effect [65].

### Process evaluation

For the process evaluation, semi-structured interviews with key figures will be typed out verbatim and analysed by two researchers independently from each other. Codes will be used to analyse the text fragments using the method of constant comparison of data extracts for each code [66]. The software Atlas-ti will be used for this purpose. The findings will be summarized in matrices, with facilitators and barriers at different levels (micro, meso, macro) and in different phases of the implementation (preparation, implementation and continuation). Descriptive analyses will be performed regarding the data on characteristics of case management (questionnaires from the case manager).

## Discussion

This study is a two year, prospective, observational, controlled, cohort study among persons with dementia and their primary informal caregiver receiving different types of case management or usual care. The study will be carried out in several regions of the Netherlands and will be accompanied by a qualitative process evaluation. Comparing two widely advocated and implemented dementia case management care models with usual care, will provide valuable information on process, clinical and cost outcomes. The extensive collection of outcomes measured, together with the naturalistic design of the cohort study and the length of follow-up are unique features of this study. This study will provide relevant information on the effect of different case management models on behaviour and quality of life in persons with dementia, on general health and burden of caregivers, and on costs and conditions for successful implementation of case management. This information endeavours to identify strengths and weaknesses of each model and may help in future policy and decision making. While various models of case management for dementia are implemented in multiple regions in the Netherlands [33], this appears to be one of the first studies to compare different case management models with usual care. As this is an observational study and treatment assignment is not randomized, bias is more likely to occur. Various forms of bias must be systematically addressed like in any prospective cohort study [67]. This includes various forms of bias from a regional level, a case management level and at an individual level.

At the regional level, confounding by socio-economic differences may limit the internal validity of the comparisons. This will be corrected for by using socio-economic characteristics of the patients (e.g. education), demographic factors (e.g. age and marital status) and by checking for clustering at the regional level in the multilevel analysis. The case management models may differ in their development (some regions started case management recently while others already exist for ten years), referral modality, the case loads of case managers and their funding, causing within and across case-management model type heterogeneity, which could have an effect on the care received by the person with dementia and their carer. A description of each model will help identify differences. The risk of selection bias potentially occurs at the patient level. Firstly, there may be confounding by indication and confounding by severity of cognitive impairments, as the likelihood of referral to case-management by a general practitioner may depend on patient characteristics, such as severity of dementia. Potential confounders include demographics like age, gender, education of patients and caregivers, time in case management, dementia severity (MMSE, dementia-type and caregiver burden (Sense of Competence questionnaire [51]). Baseline characteristics of the patients in the three intervention groups will be compared and post-hoc matching techniques and stratification will be used when necessary [39]. In a secondary analysis, semi-structured interviews with a random sample of general practitioners will be performed to identify reasons for case management referral and, thus, to identify potential selection bias. Secondly, in our opinion, there is a risk of only recruiting dementia caregivers who experience relatively less burden, limiting the external validity of the study. A non-responder analysis will be completed to identify this source of potential bias and matching techniques will be used to correct for variables such as severity of cognitive impairments (MMSE), time since diagnosis and age and health of the person with dementia and caregiver.

Attrition bias will also be analysed as an increase in severity of the disease (and therefore burden of disease). This will probably predict drop-out, leading to ‘survival’ of a relatively healthy population for the intention to treat analysis. The solution is to rigorously retrieve information on drop-outs, for example in cases where the caregiver or care receiver has died or was institutionalized. Proxy measurements will be used when the care receiver refuses or is not able to completely answer the questions in order to avoid missing data. The caregiver will act as the proxy. Proxy measurements will be used for the EQ-5D, CANE and QoL-AD. As case management is growing quickly in the Netherlands, recruiting unexposed client dyads may be a serious problem. If it appears to be impossible to recruit enough controls, then only the different case management groups will be compared against each other. A more general problem is recruiting enough people to participate, and subsequently to maintain them, in the study. As this is a burdened population that is also frequently recruited for other studies, recruiting dyads for this study is challenging and attrition rates may be high. We plan to address this in two ways. First, we must explain to participants the importance of this research as it identifies who benefits from case management and who may not and it will endeavour to determine the most efficient model. Secondly, we made every effort to avoid burdening the caregivers with unnecessary interviews or questions. An important limitation might be a relatively low participation rate that fuels confounding by indication and hinders adequate stratified analyses. As all potential participants were approached at the full time spectrum since diagnosis (except for an expected institutionalization within 6 months), an important assumption is, that the effectiveness of case management is constant over time. However, a review by Pimouguet et al. [29] found an early time effect on case management under a year and then less of an effect later on. We will handle this by using time in case management as a covariate to correct for differences between subjects in the groups. The generalizability of the results for clinicians and policy makers will largely depend on achieving the sample size required, the homogeneity of the population samples and our ability to adequately correct for potential confounders and effect modifiers in the statistical analyses. Despite the limitations addressed here, we expect that this study will provide important information on dementia case management models in The Netherlands for policy makers, health care providers, patients and informal caregivers.

## Competing interests

The authors of this paper declare that they have no competing interests.

## Authors’ contributions

JMV, LDVM, PMvdV, JEB, PvdD, FJMM, RMD, EPMvC, HEvdH, SEdR, HPJvH were all involved in the conceptual design, the manuscript revisions and approval of the final manuscript.

## Pre-publication history

The pre-publication history for this paper can be accessed here:

http://www.biomedcentral.com/1472-6963/12/132/prepub

## References

[B1] Poos MMJC Hoekstra J Slobbe LCJ Kosten van Ziekten in Nederland 2005 2008 Rijksinstituut voor Volksgezondheid en Milieu, Bilthoven

[B2] Prof Martin Prince, D.R.B.a.D.C.F., Alzheimer’s Disease International World Alzheimer Report The benefits of early diagnosis and intervention, 2011, Institute of Psychiatry 2011 King’s College London, London

[B3] International, A.s.D PMJ JJ World Alzheimer Report World Alzheimer Report 2009 Alzheimer’s Disease International,

[B4] Koopmans RT Ekkerink JL van Weel C Survival to late dementia in Dutch nursing home patients Journal of the American Geriatrics Society 2003 51 2 184 187 10.1046/j.1532-5415.2003.51056.x 12558714

[B5] Xie J Brayne C Matthews FE Survival times in people with dementia: analysis from population based cohort study with 14 year follow-up BMJ 2008 336 7638 258 262 10.1136/bmj.39433.616678.25 18187696 PMC2223023

[B6] Ministry of Health, W.a.S Guideline for Integrated Dementia Care [excerpt]: An aid for the development of integrated dementia care 2009 12

[B7] Eagles JM The psychological well-being of supporters of the demented elderly The British journal of psychiatry: the journal of mental science 1987 150 293 298 10.1192/bjp.150.3.293 3664095

[B8] Butler R The carers of people with dementia BMJ 2008 336 7656 1260 1261 10.1136/bmj.39567.647072.80 18505756 PMC2413339

[B9] Report., W.A World Alzheimer Report 2009 2009

[B10] Nederland, A Alzheimer Nederland factsheet 2010

[B11] Dunkin JJ Anderson-Hanley C Dementia caregiver burden: a review of the literature and guidelines for assessment and intervention Neurology 1998 51 1 Suppl 1 S53 S60 discussion S65-7 9674763 10.1212/wnl.51.1_suppl_1.s53

[B12] Pot AM Deeg DJH Van Dyck R Psychological well-being of informal caregivers of elderly people with dementia: changes over time Aging & Mental Health 1997 1 261 268 10.1080/13607869757164

[B13] Droes RM Effect of Meeting Centres Support Program on feelings of competence of family carers and delay of institutionalization of people with dementia Aging & mental health 2004 8 3 201 211 10.1080/13607860410001669732 15203401

[B14] Droes RM Effect of combined support for people with dementia and carers versus regular day care on behaviour and mood of persons with dementia: results from a multi-centre implementation study International journal of geriatric psychiatry 2004 19 7 673 684 10.1002/gps.1142 15254924

[B15] Acton GJ Kang J Interventions to reduce the burden of caregiving for an adult with dementia: a meta-analysis Research in nursing & health 2001 24 5 349 360 10.1002/nur.1036 11746065

[B16] Brodaty H Green A Koschera A Meta-analysis of psychosocial interventions for caregivers of people with dementia Journal of the American Geriatrics Society 2003 51 5 657 664 10.1034/j.1600-0579.2003.00210.x 12752841

[B17] Smits CH Effects of combined intervention programmes for people with dementia living at home and their caregivers: a systematic review International journal of geriatric psychiatry 2007 22 12 1181 1193 10.1002/gps.1805 17457793

[B18] Gaugler JE Unmet care needs and key outcomes in dementia Journal of the American Geriatrics Society 2005 53 12 2098 2105 10.1111/j.1532-5415.2005.00495.x 16398893

[B19] Health Council of the Netherlands Dementia, Health Council of the Netherlands 2002 Health Council of the Netherlands, The Hague 21 31

[B20] Peeters JM Informal caregivers of persons with dementia, their use of and needs for specific professional support: a survey of the National Dementia Programme BMC nursing 2010 9 9 10.1186/1472-6955-9-9 20529271 PMC2901350

[B21] van der Roest HG Validity and reliability of the Dutch version of the Camberwell Assessment of Need for the Elderly in community-dwelling people with dementia International psychogeriatrics/IPA 2008 20 6 1273 1290 10.1017/S1041610208007400 18554426

[B22] Case management Society United Kingdom What is Case management? 2011 [cited 2011 August 10, 2011]

[B23] Bodenheimer T Wagner EH Grumbach K Improving primary care for patients with chronic illness: the chronic care model, Part 2 JAMA: the journal of the American Medical Association 2002 288 15 1909 1914 10.1001/jama.288.15.1909 12377092

[B24] Bodenheimer T Wagner EH Grumbach K Improving primary care for patients with chronic illness JAMA: the journal of the American Medical Association 2002 288 14 1775 1779 10.1001/jama.288.14.1775 12365965

[B25] Boyd CM Guided care for multimorbid older adults The Gerontologist 2007 47 5 697 704 10.1093/geront/47.5.697 17989412

[B26] Brodaty H Draper BM Low LF Behavioural and psychological symptoms of dementia: a seven-tiered model of service delivery The Medical journal of Australia 2003 178 5 231 234 12603188 10.5694/j.1326-5377.2003.tb05169.x

[B27] Jansen AP Effectiveness of case management among older adults with early symptoms of dementia and their primary informal caregivers: a randomized clinical trial International journal of nursing studies 2011 48 8 933 943 10.1016/j.ijnurstu.2011.02.004 21356537

[B28] Pinquart M Sorensen S Helping caregivers of persons with dementia: which interventions work and how large are their effects? International psychogeriatrics/IPA 2006 18 4 577 595 10.1017/S1041610206003462 16686964

[B29] Pimouguet C Dementia case management effectiveness on health care costs and resource utilization: a systematic review of randomized controlled trials The journal of nutrition, health & aging 2010 14 8 669 676 10.1007/s12603-010-0314-4 PMC12880361 20922344

[B30] Schoenmakers B Buntinx F Delepeleire J Factors determining the impact of care-giving on caregivers of elderly patients with dementia. A systematic literature review Maturitas 2010 66 2 191 200 10.1016/j.maturitas.2010.02.009 20307942

[B31] Low LF Yap MH Brodaty H A systematic review of different models of home and community care services for older persons BMC health services research 2011 11 1 93 10.1186/1472-6963-11-93 21549010 PMC3112399

[B32] Schoenmakers B Buntinx F DeLepeleire J Supporting the dementia family caregiver: the effect of home care intervention on general well-being Aging & mental health 2010 14 1 44 56 10.1080/13607860902845533 20155520

[B33] Minkman MM Ligthart SA Huijsman R Integrated dementia care in The Netherlands: a multiple case study of case management programmes Health & social care in the community 2009 17 5 485 494 10.1111/j.1365-2524.2009.00850.x 19694030

[B34] Banks P Berman NPC Case management Integrating services for older people - a resource book for managers 2004 EHMA 101 112

[B35] Ligthart, S.A., Aanpak en effecten van casemanagement bij dementie. Een exploratieve studie in het kader van het Landelijk Dementieprogramma, in Kwaliteitsinstituut voor de Gezondheidszorg CBO2006, Radboud Universiteit Nijmegen

[B36] Jansen AP (Cost)-effectiveness of case-management by district nurses among primary informal caregivers of older adults with dementia symptoms and the older adults who receive informal care: design of a randomized controlled trial [ISCRTN83135728] BMC public health 2005 5 133 10.1186/1471-2458-5-133 16343336 PMC1327666

[B37] Cummings JL The Neuropsychiatric Inventory: comprehensive assessment of psychopathology in dementia Neurology 1994 44 12 2308 2314 10.1212/WNL.44.12.2308 7991117

[B38] Goldberg DP Hillier VF A scaled version of the General Health Questionnaire Psychological medicine 1979 9 1 139 145 10.1017/S0033291700021644 424481

[B39] Cummings JL The Neuropsychiatric Inventory: assessing psychopathology in dementia patients Neurology 1997 48 5 Suppl 6 S10 S16 9153155 10.1212/wnl.48.5_suppl_6.10s

[B40] Balardy L Predictive factors of emergency hospitalisation in Alzheimer’s patients: results of one-year follow-up in the REAL.FR Cohort The journal of nutrition, health & aging 2005 9 2 112 116 15791355

[B41] Bruce DG Communication problems between dementia carers and general practitioners: effect on access to community support services The Medical journal of Australia 2002 177 4 186 188 12175321 10.5694/j.1326-5377.2002.tb04729.x

[B42] Callahan CM Effectiveness of collaborative care for older adults with Alzheimer disease in primary care: a randomized controlled trial JAMA: the journal of the American Medical Association 2006 295 18 2148 2157 10.1001/jama.295.18.2148 16684985

[B43] Vickrey BG The effect of a disease management intervention on quality and outcomes of dementia care: a randomized, controlled trial Annals of internal medicine 2006 145 10 713 726 17116916 10.7326/0003-4819-145-10-200611210-00004

[B44] Hankins M The reliability of the twelve-item general health questionnaire (GHQ-12) under realistic assumptions BMC public health 2008 8 355 10.1186/1471-2458-8-355 18854015 PMC2572064

[B45] Logsdon RG Assessing quality of life in older adults with cognitive impairment Psychosomatic medicine 2002 64 3 510 519 12021425 10.1097/00006842-200205000-00016

[B46] Ware J Kosinski M Keller SD A 12-Item Short-Form Health Survey: construction of scales and preliminary tests of reliability and validity Medical care 1996 34 3 220 233 10.1097/00005650-199603000-00003 8628042

[B47] Wolfs CA Performance of the EQ-5D and the EQ-5D + C in elderly patients with cognitive impairments Health and quality of life outcomes 2007 5 33 10.1186/1477-7525-5-33 17570832 PMC1904437

[B48] Reynolds T Camberwell Assessment of Need for the Elderly (CANE). Development, validity and reliability The British journal of psychiatry: the journal of mental science 2000 176 444 452 10.1192/bjp.176.5.444 10912220

[B49] Folstein MF Folstein SE McHugh PR Mini-mental state. A practical method for grading the cognitive state of patients for the clinician Journal of psychiatric research 1975 12 3 189 198 10.1016/0022-3956(75)90026-6 1202204

[B50] Katz S Progress in development of the index of ADL The Gerontologist 1970 10 1 20 30 10.1093/geront/10.1_Part_1.20 5420677

[B51] Vernooij-Dassen MJ Assessment of caregiver’s competence in dealing with the burden of caregiving for a dementia patient: a Short Sense of Competence Questionnaire (SSCQ) suitable for clinical practice Journal of the American Geriatrics Society 1999 47 2 256 257 9988301 10.1111/j.1532-5415.1999.tb04588.x

[B52] Pearlin LI Schooler C The structure of coping Journal of health and social behavior 1978 19 1 2 21 10.2307/2136319 649936

[B53] The EuroQol Group EuroQol--a new facility for the measurement of health-related quality of life Health policy 1990 16 3 199 208 10109801 10.1016/0168-8510(90)90421-9

[B54] Brouwer WB The CarerQol instrument: a new instrument to measure care-related quality of life of informal caregivers for use in economic evaluations Quality of life research: an international journal of quality of life aspects of treatment, care and rehabilitation 2006 15 6 1005 1021 10.1007/s11136-005-5994-6 16900281

[B55] Hoeymans N van Lindert H Westert GP The health status of the Dutch population as assessed by the EQ-6D Quality of life research: an international journal of quality of life aspects of treatment, care and rehabilitation 2005 14 3 655 663 10.1007/s11136-004-1214-z 16022059

[B56] Meiland FJ Facilitators and barriers in the implementation of the meeting centres model for people with dementia and their carers Health policy 2005 71 2 243 253 10.1016/j.healthpol.2004.08.011 15607386

[B57] Dröes RM Meiland FJM Schmitz MJ Vernooij-Dassen MJFJ De Lange J Derksen E Boerema I Grol RPTM Van Tilburg W Implementation Meeting Centers Model; A Study into the Conditions for Successful Nationwide Implementation of Meeting Centers for People with Dementia and Their Carers.(In Dutch) 2003 Afdeling Psychiatrie, VU Medisch Centrum, Amsterdam

[B58] Verkade PJ Delphi research exploring essential components and preconditions for case management in people with dementia BMC geriatrics 2010 10 54 10.1186/1471-2318-10-54 20696035 PMC2928241

[B59] Dias A The effectiveness of a home care program for supporting caregivers of persons with dementia in developing countries: a randomised controlled trial from Goa, India PloS one 2008 3 6 e2333 10.1371/journal.pone.0002333 18523642 PMC2396286

[B60] ROSENBAUM PR DB RUBIN The central role of the propensity score in observational studies for causal effects Biometrika 1983 70 1 41 55 10.1093/biomet/70.1.41

[B61] Little RJ Rubin DB Causal effects in clinical and epidemiological studies via potential outcomes: concepts and analytical approaches Annual review of public health 2000 21 121 145 10.1146/annurev.publhealth.21.1.121 10884949

[B62] Burton A Billingham LJ Bryan S Cost-effectiveness in clinical trials: using multiple imputation to deal with incomplete cost data Clin Trials 2007 4 2 154 161 10.1177/1740774507076914 17456514

[B63] Efron B Missing data, imputation and the bootstrap JASA 1994 89 463 475

[B64] Black W The CE plane: a graphic representation of cost-effectiveness Med Decis Making 1990 10 212 214 10.1177/0272989X9001000308 2115096

[B65] Fenwick E O’Brien BJ Briggs A Cost-effectiveness acceptability curves–facts, fallacies and frequently asked questions Health Econ 2004 13 5 405 415 10.1002/hec.903 15127421

[B66] Miles M Huberman A Qualitative Data Analysis 1994 2 Sage Publications, Thousand Oaks, CA

[B67] Vandenbroucke JP Strengthening the Reporting of Observational Studies in Epidemiology (STROBE): explanation and elaboration Epidemiology 2007 18 6 805 835 10.1097/EDE.0b013e3181577511 18049195

